# Community-based approaches to infant safe sleep and breastfeeding promotion: a qualitative study

**DOI:** 10.1186/s12889-023-15227-4

**Published:** 2023-03-07

**Authors:** Meera Menon, Rebecca Huber, Dana D. West, Stacy Scott, Rebecca B. Russell, Scott D. Berns

**Affiliations:** 1 The National Institute for Children’s Health Quality (NICHQ), 308 Congress Street, 5th Floor, Boston, MA 02210 USA; 2 Department of Pediatrics, Warren Alpert Medical School of Brown University, Providence, RI USA; 3 Department of Health Services, Policy and Practice, Brown University School of Public Health, Providence, RI USA

**Keywords:** Infant safe sleep, Breastfeeding, Community health promotion, Perinatal education

## Abstract

**Background:**

In the U.S., sudden unexpected infant deaths (SUID) due to accidental suffocation and strangulation in bed (ASSB) are increasing, with disparities by race/ethnicity. While breastfeeding is a protective factor against infant mortality, racial/ethnic disparities are present in its uptake, and motivations to breastfeed are also often coupled with non-recommended infant sleep practices that are associated with infant sleep deaths. Combining infant safe sleep (ISS) and breastfeeding promotion on the community level presents opportunities to address racial/ethnic disparities and associated socioeconomic, cultural, and psychosocial influences.

**Methods:**

We completed a descriptive qualitative hermeneutical phenomenology using thematic analysis of focus group data. We examined the phenomenon of community-level providers promoting ISS and breastfeeding in communities vulnerable to ISS and breastfeeding disparities. We asked eighteen informants participating in a national quality improvement collaborative about i.) areas requiring additional support to meet community needs around ISS and breastfeeding, and ii.) recommendations on tools to improve their work promoting ISS and breastfeeding.

**Results:**

We identified four themes: i.) education and dissemination, ii.) relationship building and social support, iii.) working with clients’ personal circumstances and considerations, and iv.) tools and systems.

**Conclusions:**

Our findings support embedding risk-mitigation approaches in ISS education; relationship building between providers, clients, and peers; and the provision of ISS and breastfeeding supportive material resources with educational opportunities. These findings may be used to inform community-level provider approaches to ISS and breastfeeding promotion.

**Supplementary Information:**

The online version contains supplementary material available at 10.1186/s12889-023-15227-4.

## Background

**Figure 1:**
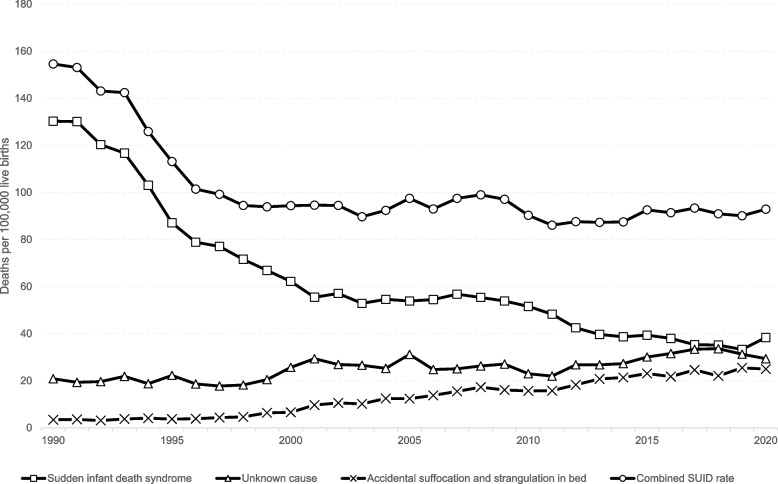
**Fig. 1 **Trends in sudden unexpected infant deaths by cause, 1990-2020. Source: Data from Centers for Disease Control and Prevention, National Center for Health Statistics. National Vital Statistics System, Mortality Files, analyzed by the National Institute for Children's Health Quality

Since the late 1990s, there has been significant progress in reducing the number of sudden unexpected infant deaths (SUID) in the U.S., yet troubling trends and disparities remain [1]. SUID encompasses deaths from sudden infant death syndrome (SIDS), accidental suffocation and strangulation in bed (ASSB), and other ill-defined and unspecified causes of infant deaths [2, 3]. Despite declining SUID, ASSB and deaths due to unknown causes are increasing (see Fig. 1), and there are substantial disparities between racial/ethnic and geographic groups [1, 3–7]. Recent rates of SUID in the U.S. are shown to be higher among non-Hispanic Black and American Indian/Alaskan Native infants compared to Hispanic, non-Hispanic White, and Asian/Pacific Islander infants [3, 4, 6–8]. Furthermore, within the U.S., there are higher rates of infant deaths in rural compared to urban communities [4, 6].

As SUID often occurs during infant sleep or in an infant’s sleeping space, there is a strong need to educate on strategies to prevent infant sleep related deaths using the American Academy of Pediatrics (AAP)‘s infant safe sleep (ISS) guidelines [9]. AAP ISS guidelines include having infants room-share with parents without bedsharing, keeping soft objects such as pillows and comforters out of the crib, and placing infants to sleep supine [10]. Despite the widespread nature of these recommendations and successful public health messaging around ISS practices to prevent SIDS and SUID, such as the Safe-to-Sleep campaign (formerly the Back-to-Sleep campaign), racial/ethnic and geographic variation in the adoption of ISS practices persist, with non-Hispanic Black parents demonstrating higher rates of non-recommended infant sleep practices compared to other groups [11, 12]. Taken together, these findings stress the need to promote ISS, particularly to prevent infant sleep related deaths among historically marginalized populations.

Despite recommendations from the World Health Organization (WHO) and the AAP encouraging infants to be exclusively breastfed for at least 6 months and up to 2 years, the U.S. ranks among the lowest in breastfeeding rates compared with other industrialized countries, and displays racial/ethnic and socioeconomic disparities in breastfeeding practices that mirror those present with SUID [13–17]. The reasons for not initiating or maintaining breastfeeding are complex and involve cultural, psychosocial, and policy-level factors, all generally leading to a lack of support for a breastfeeding individual [18–21]. In addition, factors such as preconceptions of breastfeeding in one’s social circle influence breastfeeding outcomes [16, 18, 22]. Furthermore, while breastfeeding is an important protective factor against SIDS, strong motivations to breastfeed are often coupled with non-recommended infant sleep practices such as bedsharing, and are often most pronounced among groups most vulnerable to SUID, making it challenging to rectify disparities among both ISS and breastfeeding uptake [23–28]. Thus, it is important to consider education that supports the combination of ISS and breastfeeding recommendations while accounting for parents’ contexts, preferences, and culture.

While hospital-based interventions are successful in bundling ISS and breastfeeding [29, 30], promotion in the community setting may better reach populations vulnerable to disparities [21, 24, 31–33]. For the purposes of this study, we define community as encompassing the following elements: contained by specific place-based and geographic boundaries and comprised of individuals that share social ties and connections including, but not limited to culture, socio-economic status, and race/ethnicity [34]. In a systematic review of breastfeeding interventions, Segura-Pérez and colleagues identified that policy- and community-level interventions (e.g., delivered through community agencies) were more likely to improve uptake of optimal infant care practices [21]. In addition, research depicts parents are more likely to change their behaviors and practices when they hear messages from multiple sources within their communities, underscoring the importance of delivering ISS and breastfeeding education across multiple venues [35].

Providing material resources for ISS and breastfeeding (e.g., bassinets, breast pumps), parent education, and connecting parents to supports are all successful strategies to promoting uptake of ISS and breastfeeding [31–33]. Community baby showers combine the above opportunities and demonstrate increased uptake of AAP-supported ISS practices among participants while more successfully reaching vulnerable populations compared to similar events in healthcare settings [31–33]. Further, community-driven models such as peer counseling are shown to be effective in providing breastfeeding support and enhancing breastfeeding outcomes, often more than in-hospital breastfeeding support alone [36–38]. Finally, in a community-based intervention targeting African American parents that combined ISS and breastfeeding messaging together, researchers found that participants were able to successfully maintain exclusive breastfeeding rates without bedsharing [24].

While ISS and breastfeeding interventions delivered in community settings have been successful, interventions and services may not be available in all communities [39]. Moreover, community-led work to promote ISS and breastfeeding is not immune to barriers such as the influence of parents’ social networks and cultural beliefs that can hinder uptake of recommended practices [16, 35, 40–43]. In addition, community settings are often limited by resources, such as those to train and provide continuing support for educators [44, 45].

Further, there is a dearth of research on the experiences of community-level organizations and providers in promoting both ISS and breastfeeding. Given the importance of community settings in reaching historically marginalized populations as well as coordinated messages to support ISS and breastfeeding, it is critical to understand the needs of community-level direct service providers to build systems to promote ISS and breastfeeding. Accordingly, our study examined the phenomenon of community-level providers and organizations promoting ISS and breastfeeding for their communities vulnerable to ISS and breastfeeding disparities (e.g., rural communities or those with high concentrations of Black or Indigenous populations). Specifically, our aims were: i.) to identify areas requiring additional support to meet communities’ needs around ISS and breastfeeding, and ii.) to capture recommendations and opinions on tools and resources that would improve their abilities to promote ISS and breastfeeding within their communities.

## Methods

Data collected for this analysis came from a larger evaluation of the National Action Partnership to Promote Safe Sleep Improvement and Innovation Network (NAPPSS-IIN), a multi-year project running from 2017-2022. NAPPSS-IIN aimed to make ISS and breastfeeding a national norm. Specifically, the project focused on increasing infant caregivers’ adoption of ISS and breastfeeding, as recommended by the AAP, by empowering champions within systems that serve historically marginalized families. The goal of NAPPSS-IIN was to reduce rates of SUID, increase rates of breastfeeding, and decrease disparities in these outcomes among Black and Indigenous infants. The initiative was funded by the Maternal Child Health Bureau (MCHB) of the Health Resources and Services Administration (HRSA) and led by the National Institute for Children’s Health Quality (NICHQ).

As part of the NAPPSS-IIN evaluation, in Spring 2021 NICHQ hosted a series of listening sessions held in focus groups with community-level partners working in ISS and breastfeeding promotion and participating in the NAPPSS-IIN initiative. All NAPPSS-IIN participants served communities vulnerable to ISS and breastfeeding disparities (e.g., rural communities or those with high concentrations of Black or Indigenous populations). We employed descriptive hermeneutical phenomenology as the research design [46–48]. The phenomenon studied was the lived experiences and perceptions of community-level providers promoting ISS and breastfeeding. Focus groups were selected over key informant interviews for the opportunity to foster shared discussion on the phenomenon.

We recruited a convenience sample of 18 informants participating in the NAPPSS-IIN project who consented to participate in interviews. Focus groups followed a semi-structured format and aimed to identify barriers and opportunities for promoting ISS and breastfeeding as well as to inform NAPPSS-IIN activities for the final years of the initiative. The interview guide was developed specifically for the focus groups and is available as a Supplementary Material, Additional file 1. Overall, a total of four focus groups were conducted, with the number of informants per session ranging from two to six. Table 1 provides demographics of informants’ organizations along with their roles. Focus groups were conducted virtually on the Zoom platform and included informants as well as up to four members of the research team. Transcripts from each call were obtained and transcribed using the Rev.com service. All identifying information was redacted prior to transcript review. The study protocol was reviewed and approved by Solutions Institutional Review Board.

**Table 1:** Informant roles, organizations, and service areas in focus groups

Case	Informant roles	Organization/Sector types	Service areas
Case 1	• Lactation Consultant (2) • Program Director/Manager (1) • Program Coordinator (1) • Registered Nurse (1)	• Non-profit (2) • Health system (1) • Head Start/Early Head Start (1)	• Statewide (1) • Urban (1) • Rural (1) • Unspecified service area (1)
Case 2	• Program Director/Manager (2) • Program Coordinator (1) • Clinical Consultant (1) • Data Analyst (1) • Lactation Consultant (1) • Quality Assurance Manager (1) • Registered Nurse (1)	• Department of Health (2) • Non-profit (2) • Health insurance (1) • Healthy Start (1)	• Urban (3) • Statewide (2) • Rural (1) • National (1)
Case 3	• Program Director/Manager (1) • Case Manager (1)	• Non-profit (1) • Health insurance (1)	• Statewide (1) • Urban (1) • Rural (1)
Case 4	• Program Director/Manager (2) • Program Coordinator (2) • Registered Nurse (1) • Doula (1) • Mental Health Clinician (1)	• Department of Health (3) • Non-profit (2) • Independent healthcare professional (1)	• Urban (4) • Statewide (2)

Some informants had more than one role/certification, service area, and organization/sector type

### Analysis

The research team utilized thematic analysis as the primary methodology and employed inductive coding to develop the code structure [49]. This method was used for the flexibility of approach and because limited research was available to develop a predetermined coding structure. Instead, the team developed coding and themes based on the explicit meanings emerging from the data. NVivo was used for data analysis and management [50].

To maximize trustworthiness of the analysis, investigator triangulation was used. All coders have expertise in community-based ISS and breastfeeding promotion. The researchers followed the six phases outlined by Braun and Clarke [49] which embedded reflexivity into all stages of the analysis. A combination of inductive and deductive coding was applied with initial codes based on predetermined areas of challenge and opportunity in ISS and breastfeeding promotion identified within the literature. Deductive coding was coupled with inductive coding to allow for the experience of informants to guide the domains of analysis. Initial review of two transcripts and coding were completed by MM and RH independently. Upon initial review, MM and RH met to discuss coding structure and application. This process continued in an iterative fashion until an initial codebook was established and appropriate reliability was met (pooled *K* > 0.80). RH coded the remaining two cases. A third analyst, DW, reviewed all coding applications and the codebook holistically. Disagreements were mediated with the other analysts until thematic saturation was achieved and reliability was achieved (pooled *K* > 0.80). Theme generation proceeded iteratively with the coding team discussing and refining themes independently and collaboratively. Themes were mapped onto codes to verify accuracy and exemplar quotes were identified. Memo generation and collaborative discussions around codes and themes occurred with the entire research team to maintain rigor and reflexivity.

## Results

We identified four themes discussed by community-level providers in focus groups as areas where they needed support, tools, or resources in promoting ISS and breastfeeding (Table 2).

**Table 2:** Theme and sub-theme titles, references, and exemplar quotes

Theme/Sub-theme title	N references	% references	Exemplar quote
Theme 1: Education and dissemination	271	37%	
Sub-theme 1a: Education and dissemination challenges	213	29%	“Not all nurses teach the same thing…some of them [were educated] in nursing school [which could be] 35 years ago. [At that time,] some [nurses] didn’t even [learn] information on safe sleep or, let alone, breastfeeding and safe sleep.”
Sub-theme 1b: Education opportunities	49	7%	“I think what is needed in our state is tools for open, candid conversations to talk with families about this intersection between breastfeeding and safe sleep. We see that they’re often [taught] separate but parents experience them together...And so I would like more tools that would help…to have conversations that are less prescriptive, less... preachy.”
Sub-theme 1c: Dissemination opportunities	9	1%	“[Nighttime parenting plans are] a chance to [establish], ‘Let’s walk through what’s going to happen at 2 AM and you’re exhausted and all the best intentions in the world [around ISS and breastfeeding] are gone out the window.’”
Theme 2: Relationship building and social support	252	34%	
Sub-theme 2a: Patient-provider relationship building	171	23%	“Having the Maternal Nurse Navigators that...reach out, even if it’s a small population, [to] give them information, make sure they have resources... I think that’s a really great start. I know we’re catching some of the moms [who we would normally miss].”
Sub-theme 2b: Peer-to-peer connections	81	11%	“I’ve also learned to maintain connections with mothers and families that I have served in the past because they can tell their [breastfeeding and ISS] stories to clients that I’m serving now and I think that stories are so powerful.”
Theme 3: Working with clients’ personal circumstances and considerations	144	20%	
Sub-theme 3a: Capacity	88	12%	“The biggest resource I think moms need is someone to help them, but I don’t know how you...take the load off them, because we do see...they’re just at their wits end sometimes. They’ve got so many things going on in their life, especially if they’ve got socio-economic factors affecting them.”
Sub-theme 3b: Social determinants	38	5%	“[ISS promotion makes] assumptions that [the client] has a crib or room for a crib. [But,] we don’t always know their living circumstances and how that impacts what they’re able to do”.
Sub-theme 3c: Generational barriers	18	2%	“Things have changed a lot since our clients’ mothers and grandmothers were having babies. [Extended family members will say]...‘you’ve got to give both [formula and breastmilk] because the baby is not [eating] enough,’ or, ‘you put the baby on their stomach to sleep because that’s what we did.’ We educate our moms, but then there’s that missing piece – how does it get from the mom, to the grandma, and the auntie and the older generation who did things differently?”
Theme 4: Tools and systems	71	10%	“All of our clients are Black mothers...Our initiation rate is excellent, but we run into issues [when they] go back to work. A lot of them don’t have [comprehensive] maternity leave...or they work in jobs that don’t allow them time to pump. That’s where we see the breastfeeding [rates] fall off.”
**TOTAL**	738	100%	

Exemplar quotes not provided for themes in which there were exemplar quotes from sub-themes

### Theme 1: Education and dissemination

The education and dissemination theme included challenges and opportunities around teaching, learning, and spreading information about ISS and breastfeeding promotion. Of the four themes identified, this theme was the most discussed in focus groups.

#### Sub-theme 1a: Education and dissemination challenges

Challenges to education and dissemination included an absence of effective teaching guidance (related to messaging and tone, education standards), an absence of effective messaging guidance for clients and providers (related to promoting abstinence of unsafe sleep practices, discussing ISS and breastfeeding jointly), and ignoring AAP guidelines. Thirteen informants indicated that clinician education can be dependent on when and where they received their initial training and whether they engaged in continued educational opportunities. One informant elaborated, “Not all nurses teach the same thing … some of them [were educated] in nursing school [which could be] 35 years ago. [At that time,] some [nurses] didn’t even [learn] information on safe sleep or, let alone, breastfeeding and safe sleep.” While inconsistent education standards and a lack of realistic messaging guidance were challenges to education and dissemination, nine informants shared progress and success in these areas. Informants referenced needing guidance on messaging and tone to support ISS and breastfeeding, and several shared a desire to learn best communication practices, noting how guidance on conversational approaches could result in more trusting relationships with clients. One informant elaborated on how this could enhance their ISS and breastfeeding promotion:

“I think [we could use] guidance on phrasing and wording [for] when we are providing education to [moms] postpartum or [prenatally]. How do we come about it in a non-combative or...a non-aggressive manner? Because I feel like as soon as they step into that hospital … [ISS is] being pushed down their throat.”

#### Sub-theme 1b: Education opportunities

Several informants noted that educational and dissemination challenges were often intertwined. Specifically, informants referenced ISS and breastfeeding messages as disconnected from clients’ everyday realities. An example included abstinence-based approaches to ISS, of which eleven informants were critical, noting a preference for promoting risk-mitigation. Abstinence-based ISS education was often discussed alongside the difficulty of combining ISS and breastfeeding messaging, as evidenced in the excerpt below:

“I think what is needed in our state is tools for open, candid conversations to talk with families about this intersection between breastfeeding and safe sleep. We see that they’re often [taught] separate but parents experience them together...And so I would like more tools that would help … to have conversations that are less prescriptive, less... preachy.”

#### Sub-theme 1c: Dissemination opportunities

Dissemination opportunities referred to spreading information about ISS and breastfeeding. Four informants shared examples such as helping parents with nighttime decision-making. Nighttime decision-making was discussed as a pragmatic method to prepare clients for the realities of adhering to ISS and breastfeeding practices as an overwhelmed new parent. One informant elaborated that their organization developed nighttime parenting plans in partnership with each client, which were well-received: “It’s a chance to [establish], ‘Let’s walk through what’s going to happen at 2AM and you’re exhausted and all the best intentions in the world [around ISS and breastfeeding] are gone out the window.’”

### Theme 2: Relationship building and social support

The second most discussed theme was activities that build meaningful and intentional connections for ISS and breastfeeding promotion. This theme entailed two topics: client-provider relationship building and peer-to-peer connections. While all informants agreed that these methods were positive resources to promote ISS and breastfeeding, informants also shared barriers in building relationships.

#### Sub-theme 2a: Patient-provider relationship building

Activities and meaningful connections with providers were noted as both resources and challenges to ISS and breastfeeding promotion. Informants discussed individualized attention as a key area to support uptake of breastfeeding and ISS. However, six informants discussed these techniques as subject to individual discretion. One informant shared, “[What’s lacking is] the support afterwards... [parents are] not getting lactation nurses that are supportive in the hospital. I know they have lactation consultants that … [are] there for five minutes … and then they walk out.”

Five informants successfully employed client-provider relationship building to promote breastfeeding and ISS which resulted in positive outcomes, such as reaching underserved groups, as was described by one informant:

“Having the Maternal Nurse Navigators that...reach out, even if it’s a small population, [to] give them information, make sure they have resources... I think that’s a really great start. I know we’re catching some of the moms [who we would normally miss].”

#### Sub-theme 2b: Peer-to-peer connections

Informants discussed building peer-to-peer connections in support of ISS and breastfeeding as both a resource and challenge. Examples shared included support groups, virtual/in-person events, and connections to external specialists/organizations. Some informants noted that they knew of peer-to-peer resources around ISS and breastfeeding, but that organizations may not be actively sharing this information with parents:

“It would be really nice if the hospital...[developed] a clear-cut list of community resources that mom could utilize, whether that were to be, where can they get assistance on finding a pump, [or] what support groups they have specifically for the breastfeeding community … where they have resources for in-home consultations...or for [ISS]...or to the health department to get a ‘Pack N’ Play’.”

However, the four informants who were able to broker successful peer-to-peer connections among their clients reported positive outcomes as described by the following informant:

“I’ve also learned to maintain connections with mothers and families that I have served in the past because they can tell their [breastfeeding and ISS] stories to clients that I’m serving now and I think that stories are so powerful.”

### Theme 3: Working with clients’ personal circumstances and considerations

This theme referred to clients’ experiences and/or context hindering ISS and breastfeeding guideline adoption. All informants were sympathetic to clients’ circumstances and many reported efforts to address them in service delivery. Yet, population needs continued to be a barrier to ISS and breastfeeding adoption, and fell under three topics: capacity, social determinants of health (SDOH), and generational barriers.

#### Sub-theme 3a: Capacity

Individual conditions that hampered ISS and breastfeeding adoption included client overwhelm which often led to clients following their intuition around ISS and breastfeeding. Thirteen informants expressed sensitivity to the overwhelming nature of new parenthood and impacts on breastfeeding and ISS. Few informants were able to offer practical solutions to address overwhelm beyond relationship building, as discussed below:

“The biggest resource I think moms need is someone to help them, but I don’t know how you...take the load off them, because we do see...they’re just at their wits end sometimes. They’ve got so many things going on in their life, especially if they’ve got socio-economic factors affecting them.”

Six informants referenced how parents may rely on their own instincts during duress, which may not align with ISS and breastfeeding guidelines. The main mechanism shared by four informants to remedy this dynamic was social support, as evidenced by this quote:

“When it comes to … bed sharing, a lot of times parents know it’s a risk … but they do it anyway because...it’s easy for them. There’s this fine line that you walk sometimes, because...We don’t want to make them feel bad or guilty, but...you also want to provide them with truth and research and information.”

#### Sub-theme 3b: Social determinants

Nine informants discussed broader conditions that hindered ISS and breastfeeding adoption such as opioid use, poverty, mental health, and language barriers. As with capacity, informants expressed sensitivity on these topics and took efforts to address SDOH in their work. Yet, informants reported continued structural barriers to sufficiently address SDOH while promoting ISS and breastfeeding. One informant expressed how their ability to promote ISS was limited by SDOH: “[ISS promotion makes] assumptions that [the client] has a crib or room for a crib. [But,] we don’t always know their living circumstances and how that impacts what they’re able to do.”

#### Sub-theme 3c: Generational barriers

ISS and breastfeeding experiences differing from current recommendations were discussed by four informants as hindering ISS and breastfeeding adoption. Informants noted the influence of cultural/family practices on ISS and breastfeeding adherence. While some sought to engage all extended family members who interact with a newborn to remedy this barrier, they reflected on their limited capacity to do so, as evidenced in the quote below:

“Things have changed a lot since our clients’ mothers and grandmothers were having babies. [Extended family members will say]...‘you’ve got to give both [formula and breastmilk] because the baby is not [eating] enough,’ or, ‘you put the baby on their stomach to sleep because that’s what we did.’ We educate our moms, but then there’s that missing piece – how does it get from the mom, to the grandma, and the auntie and the older generation who did things differently?”

### Theme 4: Tools and systems

Informants shared specific and actionable instruments and infrastructure as mechanisms for ISS and breastfeeding promotion. These mechanisms were framed by informants as components working together or failing to work together to promote ISS and breastfeeding. Seven informants found material resources such as “Pack N’ Plays”, bassinets, and baby gates helpful in promoting ISS and breastfeeding. One informant noted how the presence of material resources in the home could “bring on [important] conversations in the community” around ISS and breastfeeding when friends and family members visited.

Informants discussed different media portrayals of ISS and breastfeeding including videos, pamphlets, and literature with positive and negative sentiments. While some reported their organization’s educational videos had potentially shaming messaging, others found their videos effective in promoting ISS and breastfeeding. Additionally, though some informants found providing pamphlets and literature on ISS and breastfeeding to be helpful, others were concerned about overwhelming clients with too much information.

Policies and work environments were referenced as mutually reinforcing mechanisms for ISS and breastfeeding promotion. Three informants reported that in the absence of comprehensive paid family leave in their state, parents returned to work earlier than they would have liked, and that work environments did not promote ISS and breastfeeding. One informant indicated that, “working in jobs that don’t allow them time to pump [is] where we see breastfeeding [rates] fall off after those first couple of months” within their community.

## Discussion

Community-based approaches demonstrate promise in promoting ISS and breastfeeding among underserved populations [21, 24, 31–33]; however, study informants noted substantial barriers to ISS and breastfeeding promotion within their communities, all of which were vulnerable to ISS and breastfeeding disparities (e.g., rural communities or those with high concentrations of Black or Indigenous populations). Despite the challenges, informants also highlighted opportunities to promote ISS and breastfeeding practices in their communities. Our findings suggest that addressing the challenges informants identified and bolstering work in areas deemed promising would help community-level organizations increase the reach of ISS and breastfeeding promotion.

### Challenges and opportunities related to education and dissemination

Education and dissemination around ISS and breastfeeding guidelines was discussed by most informants as a challenge; however, these concepts were also referenced as areas of opportunity. Providers shared difficulties in educating parents on effective ISS and breastfeeding strategies, mostly referring to tension around abstinence-based ISS education. Bedsharing is often the reality for many families as a sizeable number of parents will unintentionally bedshare [51], and many parents with strong breastfeeding intentions bedshare [23, 26, 28]. Study informants noted the struggle of not engaging in conversations around bedsharing in their communities, especially as abstinence-based approaches to ISS education may deter parents from initiating breastfeeding or can serve to prematurely end breastfeeding [52].

The Academy of Breastfeeding Medicine highlights the need to promote recommended ISS guidelines while incorporating messaging on risk-mitigation [52, 53]. These approaches holistically consider factors that may lead to risk of infant death (i.e., smoking, parent consumption of alcohol or drugs, and prematurity/low birth weight) and encourage providers to engage in conversation around ISS while addressing strategies to reduce risk of adverse outcomes should unsafe sleep practices occur [52, 53]. Risk-mitigation approaches use flexible, stigma-free methods that allow for incremental changes to move towards ISS rather than parents feeling overwhelmed by fully overhauling practices [52]. Indeed, some study informants noted success in the provision of risk-mitigation support and education, especially within the context of parents’ nighttime decision-making. Providing resources around risk-mitigation to a broader swath of providers, including those working on the community level, may rectify some tensions around ISS education. In this manner, providers may still support ISS practices as endorsed by the AAP while meeting parents where they are.

Another challenge related to education and dissemination was variation in community-level providers’ education around ISS and breastfeeding and training on sensitive message delivery. While studies depict barriers for paraprofessional and community-focused education models on recommended ISS practices [44], efforts to train providers on education strategies improve client outcomes [45, 54]. Providing ISS and breastfeeding training opportunities for paraprofessionals [45] as well as cultural sensitivity trainings targeted on message dissemination [54] enhance the quality of support clients receive and their ISS and breastfeeding outcomes [45, 54]. Taken together, the challenges noted by our study informants underscore the need for continued education and support for community-level providers around educating and messaging current ISS and breastfeeding guidelines.

### Challenges and opportunities related to relationship building and social support

Informants noted their capacity to build relationships and provide client support both directly and through peers was a barrier to promoting ISS and breastfeeding in their communities. However, embedded in these challenges were opportunities to strengthen community-led promotion. Study informants shared that clients found current interactions with providers lacking and preferred additional opportunities for connection. Parents look to healthcare providers as trusted sources of information; yet, many parents are conflicted about querying providers on infant care practices, noting they do not want to bother clinicians [40]. Moreover, research finds that minoritized parents feel they are overlooked or forgotten by providers and city services [43]. Providers, in turn, mention time limitations for client connections and education deter them from engaging in conversations around ISS practices [45].

These challenges underscore that concerted strategies must be taken to build connections within parents’ routine interactions with providers. Having providers engage in conversational, shared decision-making approaches to promoting ISS and breastfeeding could assist in fostering relationship building. Conversational approaches around infant care, particularly on ISS and breastfeeding, are positively associated with client uptake of these practices [55–58]. With informants highlighting relationship building between providers and clients as an area of need, providing trainings and resources to community-level providers around shared decision-making could support efforts to promote ISS and breastfeeding.

Similarly, peer-to-peer connections were discussed as a challenge to ISS and breastfeeding promotion among study informants who noted their limited ability to facilitate connections within their programs. However, several informants shared preliminary efforts around supporting peer-to-peer connections, remarking that these relationships may support ISS and breastfeeding practice uptake. Parents utilize peer networks, including social media, as a source of information, support, and bonding regarding infant care [35, 40, 42]. Yet, peer-to-peer spaces have less formalized content moderation than those maintained by professionals, providing an area of opportunity for community-level organizations to train and engage peer counselors as moderators. Substantial research depicts that peer counselor support is associated with uptake of optimal infant care practices as counselors may provide individualized support for parents [36–38, 59]. Building on opportunities identified by our study informants, training peer counselors and facilitating connections among peers may provide another avenue to support uptake of ISS and breastfeeding.

### Challenges and opportunities related to working with clients’ personal circumstances and considerations

Working with clients’ personal circumstances and considerations were barriers for informants to promote ISS and breastfeeding. These circumstances were primarily related to parents’ capacity, generational barriers, and SDOH. Informants shared that when parents’ capacities were limited, parents could revert to their own instincts regarding ISS and breastfeeding practices. Other research demonstrates that parental life circumstances tend to dictate decisions around ISS and breastfeeding [18, 27, 40, 42, 43]. For instance, social and physical support at home is strongly associated with the uptake of breastfeeding and ISS [16, 18, 27, 35]. Further, mental health, poverty, and substance abuse may drive parental decisions around the uptake of ISS and breastfeeding practices. For example, conditions related to poverty and trauma can inform decisions around infant care [27, 60]; trauma histories are related to lower breastfeeding initiation [60] and in certain housing contexts, it may be safer for parents to sleep with their infants, rather than recommended ISS practices [27].

Study informants also noted that generational barriers influencing parents’ decision making as a challenge in their communities. Research shows that certain African American and American Indian/Alaskan Native groups may be aware of ISS guidelines but prefer to follow infant sleep practices that are deemed “unsafe” due to cultural preferences, making it challenging for providers to engage in conversations around shifting behavior [22, 43, 61, 62]. These challenges may be due to generational barriers, as American Indian/Alaskan Native parents and African American parents often look to older family members, who may be skeptical of recommendations on ISS and breastfeeding, as trusted sources around infant care [18, 22, 40, 41, 43, 63]. With research depicting that parents vulnerable to disparities in ISS and breastfeeding value grandparents and elders’ opinions on infant care, and that grandparents and elders tend to be wary of infant care recommendations that run counter to cultural beliefs [18, 22, 27, 63], it is critical that providers consider culturally congruent care in building relationships with families.

### Challenges and opportunities related to tools and systems

The last barrier study informants shared was regarding tools and systems, such as media messaging, material resources, and societal factors supporting the uptake of ISS and breastfeeding in their communities. Media portrayals were noted as an area of challenge for informants, who discussed that ISS and breastfeeding messaging could be shaming. Research depicts parents’ struggle to negotiate messaging related to infant care from educational resources and health campaigns with information received from friends and family [22, 43, 63]. In addition, media portrayals of ISS and breastfeeding are not often culturally congruent in delivery and intended target population [22, 43]. As parents are more likely to change their behavior related to infant care when messaging received is additive and not contradictory [35], it is important to ensure that media resources and messaging convey aligned information.

Informants noted that the provision of material resources served as an opportunity to promote ISS and breastfeeding. Material resources such as portable cribs were discussed as an entry point to engage in conversations about ISS and breastfeeding with clients. Prior studies find that events such as community baby showers effectively reach groups that are vulnerable to ISS and breastfeeding disparities [31–33]. In addition, the activities conducted at community baby showers—providing physical and educational resources while connecting parents with providers in their communities—have demonstrated success in increasing the uptake of ISS [31–33]. Given the opportunities noted by our study informants, supporting activities that distribute material resources and promote social connections and education could ultimately increase uptake of ISS and breastfeeding.

Finally, informants shared societal factors such as clients’ inability to take parental leave as hindering uptake of recommended ISS and breastfeeding practices. Research depicts policy-level, community-focused interventions improve adherence to of ISS and breastfeeding compared to initiatives that address individual-level behavior [21]. In an analysis of policies protecting and supporting breastfeeding in the workplace, researchers found that worker protection laws such as providing space to breastfeed and mandating breaks to facilitate breastfeeding and pumping benefited Hispanic and African American women more so than other groups [21]. As breastfeeding decision-making is strongly related to ISS behavior [23], policies providing supportive structures and environments related to breastfeeding may work to promote the uptake of both recommended ISS and breastfeeding practices.

### Limitations and future directions

While our findings have implications for providing support and promoting innovative approaches to community-level promotion of ISS and breastfeeding, our results must be interpreted with the following considerations. Firstly, all focus groups were conducted virtually using the Zoom platform due to the COVID-19 pandemic, which may have hindered our ability to capture non-verbal communication from informants. We were also unable to examine whether responses differed based on demographics of our study informants as well as the communities they serve. Future research may consider exploring similarities and differences shared by community-level providers in promoting ISS and breastfeeding based on demographic factors. In addition, we broadly categorized community-level organizations and providers for inclusion in our study, meaning informants’ organizations could target either individual-level or policy-level issues. Additional research may consider exploring whether organization needs differ based on their target populations and scope. Moreover, our analysis did not include the perspective of clients. To develop a holistic perspective of community needs around ISS and breastfeeding support, future studies may examine the concordance between provider and client responses. Additionally, researcher bias is a possibility in qualitative research; we took efforts to minimize bias by discussing discrepancies with a third coder and using investigator triangulation. Finally, qualitative research by nature cannot determine the prevalence of each opinion shared in focus groups.

## Conclusions

Given the disparities that exist in ISS and breastfeeding practice uptake in the U.S. [11–13, 15], advocates look to the community setting to reach families vulnerable to disparities. However, the unique considerations of providers and organizations that work within community-based settings have not been thoroughly explored. Accordingly, our study is among the first to qualitatively identify community-level provider and organizational needs to effectively promote both ISS and breastfeeding. Informants in our study underscored challenges such as: navigating abstinence-based approaches in ISS education, while simultaneously promoting breastfeeding [51–53]; limited support to build meaningful connections between providers, clients, and peers [40, 45]; parents’ capacities, cultural contexts, and ability to access material resources [18, 27, 40, 43]; and the broader systems that serve as barriers to ISS and breastfeeding adherence [21, 22, 43]. Nonetheless, study informants also noted areas of innovation and opportunity including: embedding risk-mitigation and conversational approaches in ISS education [52, 53]; supporting relationship building and peer to peer support [36–39]; and combining the provision of material resources to support ISS and breastfeeding with education [31–33]. By supporting community-level providers and organizations in addressing their unique challenges as well as building upon their successes and innovations, ISS and breastfeeding advocates may improve ISS and breastfeeding outcomes among historically marginalized populations.

## Supplementary Information

**Additional file 1.**

## Data Availability

Audio transcriptions and recordings are not publicly available to protect informant privacy. Data for analysis were de-identified and are available upon reasonable request.

## Abbreviations

AAP: American Academy of Pediatrics
ASSB: Accidental suffocation and strangulation in bed
HHS: Department of Health and Human Services
HRSA: Health Resources and Services Administration
ISS: Infant safe sleep
MCHB: Maternal Child Health Bureau
SDOH: Social determinants of health
SIDS: Sudden infant death syndrome
SUID: Sudden unexpected infant death
WHO: World Health Organization
