# Supplementary material for: Community-based approaches to infant safe sleep and breastfeeding promotion: a qualitative study
Source: BMC Public Health. 2023 Mar 7;23:437. doi: 10.1186/s12889-023-15227-4 (PMC9989577; doi:10.1186/s12889-023-15227-4)
Supplement: Supplementary file 1 — Additional file 1. [file 12889_2023_15227_MOESM1_ESM.docx]

**NAPPSS-IIN Focus Group Interview Guide**

**Participants and roles**

- Facilitator (TBD prior to call)
  - Member of NAPPSS-IIN team; leads discussion
- Note-taker (TBD prior to call)
  - Member of NAPPSS-IIN team; assists in leading discussion; can ask questions for clarifying notes
- NAPPSS-IIN team members listening in (TBD prior to call)
  - Participates in introductions at start of the call (introduces role on NAPPSS-IIN project; why they are listening into the discussion); does not interrupt discussion after introduction
- Listening session participants
  - Participates in discussion

**Protocol**

***Facilitator****:* Hello, everyone. Thank you for your time and agreeing to participate in this listening session. Before we get started, I’d like to do introductions so we can all get to know each other on this call. My name is [name] and I am a [role at NICHQ]. I also want to briefly introduce the NAPPSS-IIN project, which stands for the National Action Partnership to Promote Safe Sleep Improvement and Innovation Network. NAPPSS-IIN is led by the National Institute for Children’s Health Quality (NICHQ) and funded by the Maternal and Child Health Bureau (MCHB) of the Health Resources and Services Administration (HRSA). The purpose of NAPPSS-IIN is to make the safe sleep and breastfeeding recommendations as supported by the AAP a national norm. And I will pass over to Notetaker to introduce themself.

***Notetaker****:* Hello, my name is [name] and I am a [role at NICHQ] on the NAPPSS-IIN project. I will be taking notes on today’s call. I may ask a few clarifying questions.

***NAPPSS-IIN team members listening in****:* Hello, my name is [name] and I am a [role at NICHQ] on the NAPPSS-IIN project. I will be listening in on today’s call.

[Pause for remainder of participants to introduce themselves]

***Facilitator****:* Great, again, I want to thank you all for your time today and agreeing to participate in this listening session. The purpose of this discussion is to talk about the opportunities for and challenges of promoting safe-sleep and breastfeeding for families.

This information will help guide the NAPPSS-IIN team as we aim to support community-driven organizations in promoting safe sleep and breastfeeding among the families they serve. Therefore, we are hoping that you can elaborate during our discussion today on one main question: What would you like to improve upon in your work to promote safe sleep and breastfeeding for the families you serve? In addition, what tools, resources, or supports do you need to improve your work?

[Allow participants to respond; if there is difficulty moving the conversation, use below prompts]

**Prompts to move discussion**

- You shared some of the successes [*reference successes participants shared in survey prior to listening session]* that you have had in promoting safe sleep and breastfeeding in your community with us prior to the call. Can you elaborate on these areas? What could help you continue to improve upon this work?
  - Based on the successes you shared, what recommendations would you have for other organizations who would like to adapt your strategies?
- You shared some of the challenges [*reference challenges participants shared in survey prior to listening session]* that you have had in promoting safe sleep and breastfeeding in your community with us prior to the call. Can you elaborate on these areas? What would help you move past these barriers?
- What issues do families in your community say are standing in the way of them practicing safe sleep and breastfeeding? What does your organization need to help families address these issues?
- What resources, supports, or tools have you used in the past that have helped you to promote safe-sleep and breastfeeding among families? What did you like, and did you not like about these resources? What would have made them better?
- What recommendations do you have for us to support community-driven organizations as they promote safe sleep and breastfeeding practices to the families they serve?

***Facilitator****:* Thank you all for sharing your experiences, ideas, and learnings with us today. The information that you shared is extremely important to us as we develop Cohort C, the next iteration of the NAPPSS-IIN project.

We will use your insights to make sure that we are doing the best to support the project and the community teams that will participate. As a token of our gratitude for your time, you will be receiving a $25 dollar Visa gift-card emailed directly to you. Please be on the lookout for that gift card over the next few days. Do not hesitate to reach out to the NAPPSS-IIN team if you have any questions or thoughts following this focus group. We will include the NAPPSS-IIN email in the chat. Once again, thank you so much for your time today!
